# Core RNAi Machinery and *Sid1*, a Component for Systemic RNAi, in the Hemipteran Insect, *Aphis glycines*

**DOI:** 10.3390/ijms14023786

**Published:** 2013-02-08

**Authors:** Raman Bansal, Andy P. Michel

**Affiliations:** Department of Entomology, Ohio Agricultural Research and Development Center, The Ohio State University, 1680 Madison Ave., Wooster, OH 44691, USA; E-Mail: bansal.67@osu.edu

**Keywords:** insect RNAi, aphid, *Aphis glycines*, *Dicer*, *Argonaute*, *R2D2*, *Sid1*, gene expression

## Abstract

RNA interference (RNAi) offers a novel tool to manage hemipteran pests. For the success of RNAi based pest control in the field, a robust and systemic RNAi response is a prerequisite. We identified and characterized major genes of the RNAi machinery, *Dicer2* (*Dcr2*), *Argonaute2* (*Ago2*), and *R2d2* in *Aphis glycines*, a serious pest of soybean. The *A. glycines* genome encodes for at least one copy of *Dcr2*, *R2d2* and *Ago2*. Comparative and molecular evolution analyses (d*N*/d*S*) showed that domain regions of encoded proteins are highly conserved, whereas linker (non-domain) regions are diversified. Sequence homology and phylogenetic analyses suggested that the RNAi machinery of *A. glycines* is more similar to that of *Tribolium casteneum* as compared to that of *Drosophila melanogaster*. We also characterized *Sid1*, a major gene implicated in the systemic response for RNAi-mediated gene knockdown. Through qPCR, *Dcr2*, *R2d2*, *Ago2*, and *Sid1* were found to be expressed at similar levels in various tissues, but higher expression of *Dcr2*, *R2d2*, and *Ago2* was seen in first and second instars. Characterization of RNAi pathway and *Sid1* in *A. glycines* will provide the foundation of future work for controlling one of the most important insect pests of soybean in North America.

## 1. Introduction

Transgenic crops have emerged as an effective tool for global insect pest management. Plants expressing cry-toxin encoding genes from the bacterium *Bacillus thuringienesis* (*Bt*) have achieved tremendous success for managing various lepidopteran and coleopteran pests [1,2]. However, *Bt*-based transgenic crops are not effective against hemipteran crop pests, which include aphids, leafhoppers, and whiteflies [3–5]. The inefficacy of *Bt* toxins to hemipteran insects has been attributed to various factors [6–11] but the exact cause remains unclear, thus providing difficult challenges for managing this group of economically important crop pests.

Employing RNA interference (RNAi) offers an alternative method for hemipteran pest control, and is expected to achieve the same level of success as *Bt*-based transgenic crops [12]. RNAi is based on the introduction of dsRNA which, after processing, binds to the mRNA transcript of the target gene [13]. For RNAi-based crops to be successful, the target insect must express the RNAi pathway as well as a systemic response that leads to dsRNA processing and mRNA degradation in several tissues, not just the midgut where dsRNA uptake occurs [14]. In the absence of proper genetic machinery or systemic RNAi, the resulting knockdown will have either no effect or be localized (which may or may not cause mortality).

Two parallel and closely related RNA pathways exist in eukaryotes: RNAi (also referred to as small interfering, siRNA) and micro-RNA (miRNA) pathways [15]. Both plants and invertebrates use the RNAi pathway as an innate immune system against viruses [16–18]. Alternatively, the miRNA pathway is triggered by small non-coding RNAs (ncRNAs) that regulate gene expression (reviewed in [19]). Despite their different roles in cellular processes, similar proteins are used. The proteins Dicer1, Loquacious and Ago1 are involved in the miRNA pathway whereas Dicer2, R2d2 and Ago2 are involved in the RNAi pathway. Previous investigations of the miRNA pathway genes in the pea aphid (*Acyrthosiphon pisum*) revealed the presence of duplications as well as positive selection and rapid evolutionary divergence [20]. While no duplications were found in RNAi pathway genes, no molecular or comparative genomic characterization was provided. In terms of RNAi-based control, the sequence characterization and gene expression of the RNAi pathway genes in aphids is necessary.

In insects, the red flour beetle, *Tribolium castaneum*, has emerged as the model for systemic RNAi [21,22]. However, unlike *T. castaneum*, *Drosophila melanogaster* is not very sensitive to systemic RNAi. This difference could possibly due to the gene called *systemic RNA interference deficient-1* (*Sid1*) [23,24]. While the *T. casteneum* genome encodes for three *Sid1* like genes, *Sid1* homologs are absent in *D. melanogaster*. The *Sid1*-encoded protein is responsible for spreading the amplified signal for RNAi [25]. Studies on insect *Sid1* genes are preliminary, although homologs can be found in other insect taxa [26–28]. Development of RNAi-based insect control, especially for non-model insects such as aphids, can be expedited through the understanding of RNAi pathway, including evidence for a systemic response. For example, the absence of *Sid1*, or its weak levels of expression in multiple tissues, would indicate a lack of systemic RNAi.

*A. glycines* is a major pest of soybean throughout soybean-growing regions in North America [29], causing yield losses as high as 40%. Soybean producers have adopted regular scouting and insecticidal sprays as part of their management practices, which eventually have led to a significant economic impact on soybean production [30]. Several soybean genes have been identified that provide some level of resistance to *A. glycines* [31]. However, *A. glycines* has already overcome this resistance [32], threatening the utility and sustainability of this management tactic. Novel strategies are needed to control this pest, including RNAi-based insect resistance.

Our goal, therefore, was to provide a more comprehensive characterization of the aphid RNAi machinery, including comparisons of sequence evolution and gene expression across multiple tissues. Characterization and understanding of the RNAi machinery will help develop and improve robust RNAi methods for gene knockout in *A. glycines*. In the current study, we have identified and characterized genes encoding the major components of RNAi machinery, specifically Dcr2, Ago2, and R2d2 from a transcriptome database [33]. Using comparative genomic, molecular evolution and phylogenetic approaches, we determined evolutionary relationships among the hemipteran RNAi genes and those from other insect orders. We also characterized *Sid1*, which could possibly be involved in a systemic response for RNAi-mediated gene knockdown. Additionally, we compared the expression of these genes in multiple developmental stages and tissues to evaluate the RNAi pathway activity.

## 2. Results

### 2.1. Identification of Core RNAi Machinery and a Factor for Systemic RNAi

A search for major components of RNAi machinery in an *A. glycines* transcriptomic database revealed that its genome encodes for at least one copy of *Dcr2*, *R2d2* and *Ago2* (Table 1) as well as the cDNA of a putative factor for systemic RNAi, *Sid1*. The proteins encoded by *AyDcr2*, *AyR2d2*, *AyAgo2*, and *AySid1* have a high level of sequence similarity with corresponding proteins of various insects and, as expected, their closest matches were other Aphididae species, *A. pisum* or *Aphis gossypii*.

#### 2.1.1. Characterization of *AyDcr2*

**Figure 1:**
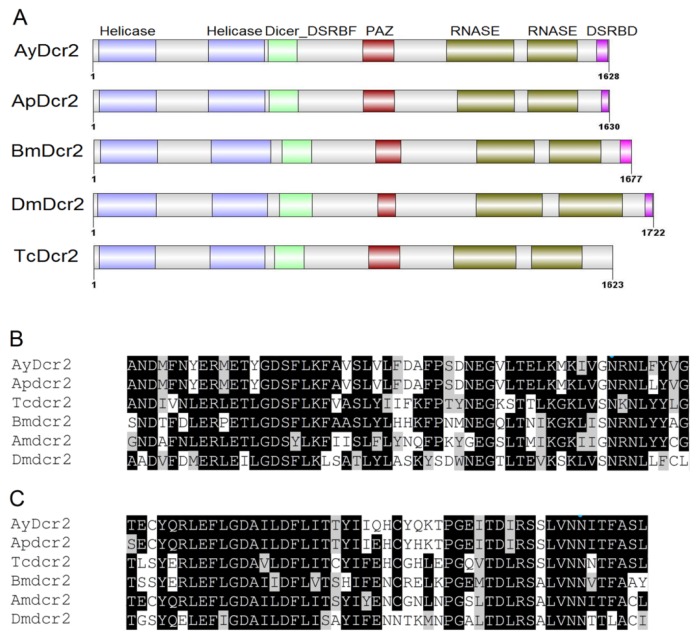
(**A**) Domain architecture of Dicer2 proteins from various insects. (**B**) and (**C**) Partial alignment of RNAse IIIa (**B**) and RNAse IIIb (**C**) domain sequences from insect Dcr2 proteins. The conserved and similar amino acid residues are labeled in black and grey backgrounds respectively. Dicer2 proteins were from *Acyrthosiphon pisum* (Ap), *Aphis glycines* (Ay), *Apis mellifera* (Am), *Bombyx mori* (Bm), *Drosophila melanogaster* (Dm), and *Tribolium castaneum* (Tc).

Structural analysis of AyDcr2 revealed the presence of all signature domains of Dcr2 proteins (Figure 1A). It contained two amino-terminal DExH helicase domains, a Dicer double-stranded RNA-binding fold (Dicer_DSRBF) domain, a PAZ domain, two RNase III domains and a carboxy-terminal dsRNA binding (DSRBD) domain. The domain architecture of Dcr2 proteins from various insects is identical, except for the absence of a DSRBD domain in Dcr2 of *T. castaneum*. However, there are conserved amino-acid residues in the corresponding region of Dcr2 in *T. castaneum* [26]. For a particular domain in insect Dcr2 proteins, ScanProsite attributed a similar profile hit score (Table S1). The RNase III domains possess endonuclease enzymatic activity that results in processing of dsRNA into siRNA. The multiple sequence alignments revealed a conserved nature of both RNase III domains in various insects (Figures 1B,C and S1). The RNase IIIa and RNase IIIb of AyDcr2 shared identities to those of Dcr2 in *T. castaneum* (36.0% and 51.6% respectively) and *D. melanogaster* (24.6% and 38.8% respectively).

#### 2.1.2. Characterization of *AyR2d2*

**Figure 2:**
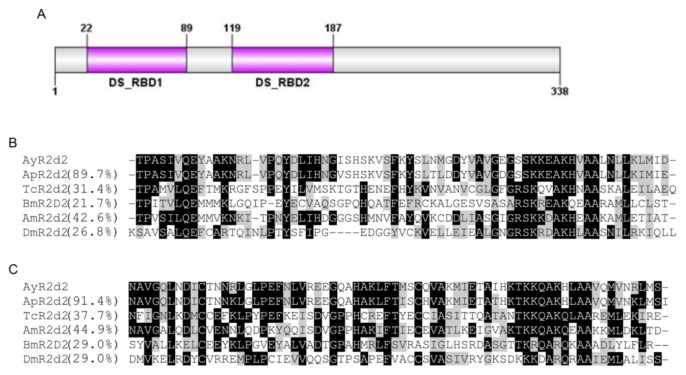
(**A**) Domain architecture of R2d2 in *A. glycines*. Both DS_RBD1 and DS_RBD2 domains are indicated; (**B**) and (**C**) Multiple sequence alignment of DS_RBD1 (**B**) and DS_RBD2 (**C**) domain sequences from insect R2d2 proteins. The conserved and similar amino acid residues are labeled in black and grey backgrounds respectively. The figures in the parentheses indicate the percent identity to the respective domain of AyR2d2. R2d2 proteins were from *Acyrthosiphon pisum* (Ap), *Aphis glycines* (Ay), *Apis mellifera* (Am), *Bombyx mori* (Bm), *Drosophila melanogaster* (Dm), and *Tribolium castaneum* (Tc).

The domain analysis at ScanProsite revealed that R2d2 (AyR2d2) contained 2 characteristic double-stranded RNA binding domains (DS_RBD1 and DS_RBD2) at the amino-terminal (Figure 2A). Both DS_RBDs are critical for binding of dsRNA to R2d2. At the start of RNAi process, dsRNA binds to DS_RBD1. After cleavage by Dicer-2, siRNAs are released from DS_RBD1 and bind to DS_RBD2 of R2d2 [34–36]. A multiple sequence alignment indicates only a small degree of sequence similarity among various insect R2d2s (Figure S2). However, both DS_RBDs are highly conserved (Figure 2B,C). The DS_RBD1 of AyR2d2 shared an identity of 31.4% and 26.8% to those of R2d2 in *T. castaneum* and in *D. melanogaster*, respectively. Similarly, DS_RBD2 of AyR2d2 shared an identity of 37.7% and 29.0% to those of R2d2s in *T. castaneum* and in *D. melanogaster*, respectively.

#### 2.1.3. Characterization of *AyAgo2*

**Figure 3:**
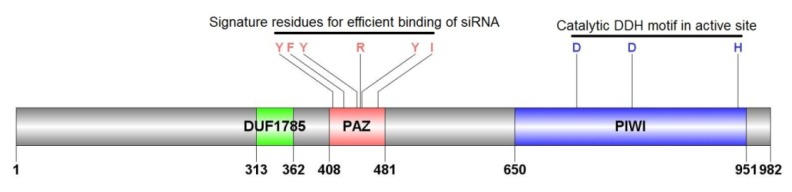
Ago2 protein in *A. glycines*. AyAgo2 is 982 amino acids in length. The relative positions of three domains (domain of unknown function (DUF1785), PAZ, and PIWI) are shown.

The AyAgo2 protein contained three characteristic domains which are domain of unknown function 1785 (DUF1785), PAZ domain and PIWI domain (Figure 3A). The PAZ domain contained signature residues that are required for efficient binding of siRNA [37]. These residues are highly conserved in various organisms. The PIWI domain contained the signature DDH motif that forms the active site of Ago2 proteins [38]. The multiple sequence alignment indicated that all 3 domains show high level of conservation in Ago2 proteins of various insects (Figure S3). However, linker (non-domain) regions are not conserved.

#### 2.1.4. Characterization of *AySid1*

**Figure 4:**
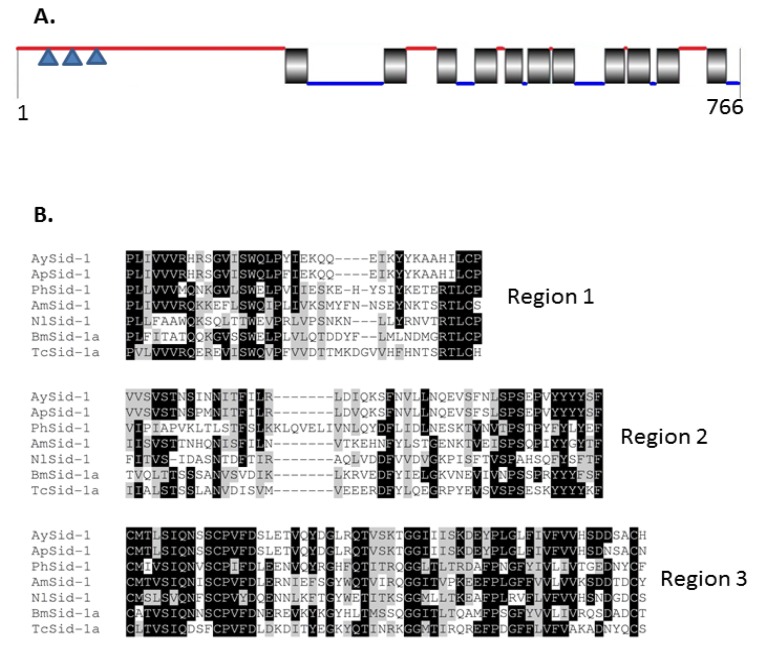
(**A**) Sid1protein architecture in *A. glycines*. AySid1 is 766 amino acids in length. Red and blue lines indicate extracellular and intracellular domains respectively. Vertical black boxes indicate transmembrane domains. Inverted blue triangles indicate approximate locations of conserved regions 1–3 (see Figure 4B) (**B**) Conservation in long extracellular domain at amino-terminal of Sid1 proteins. Three highly conserved regions in extracellular domain at amino-terminal of Sid1 protein in various insects are shown. Regions 1 and 3 are conserved in vertebrates and nematodes [26]. Sid1 proteins were from *Acyrthosiphon pisum* (Ap)*, Aphis glycines* (Ay), *Apis mellifera* (Am), *Bombyx mori* (Bm), *Nilaparvata lugens* (Nl), *Pediculus humanus* (Ph), and *Tribolium castaneum* (Tc).

At the *N*-terminus, AySid1 was predicted to contain a secretion signal peptide of 17 amino acids (Figure S4). Scanning of the AySid1 sequence using the TMHMM Server v. 2.0 indicated a long extracellular *N*-terminal (284 aa) which is followed by 11 hydrophobic, transmembrane α-helices (Figure 4A). Presence of longer extracellular domain at amino terminal is a characteristic feature of Sid1 proteins in various organisms. In insects, this domain contains 3 regions of highly conserved residues (Figure 4B). Moreover, regions 1 and 3 are conserved even between insects, nematodes and vertebrates [26]. Multiple sequence alignment of Sid1 like proteins from various insects also suggested a high level of sequence conservation at the carboxy terminal (Figure S5).

### 2.2. Phylogenetic Analysis of AyDcr2, AyR2d2, AyAgo2, and AySid1

#### 2.2.1. Dcr2

**Figure 5:**
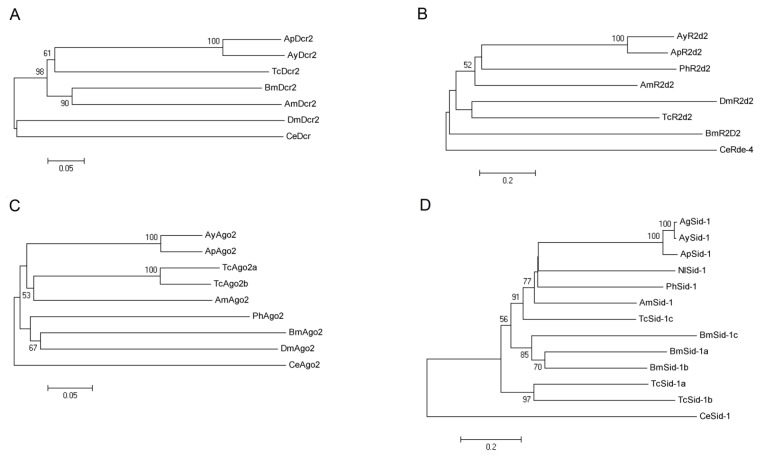
Phylogeny of insect Dcr2, R2d2, Ago2 and Sid1 proteins. The phylogenetic trees were constructed from amino acid sequences corresponding to RNAse IIIa, DS_RBD1, and PIWI domains of insect Dcr2 (**A**), R2d2 (**B**), and Ago2 (**C**) respectively. Full length protein sequences were used to construct the phylogenetic tree for Sid1 (**D**). The percentages of replicate trees in which the sequences clustered together in the bootstrap test (10000 replicates) are shown (only above 50%) next to the branches. The tree is drawn to scale, with branch lengths in the same units as those of the evolutionary distances used to infer the phylogenetic tree. The scale bar represents 0.2 (**B** and **D**) or 0.05 (**A** and **C**) expected substitutions per amino acid position. Various proteins for phylogenetic analysis were from *Acyrthosiphon pisum* (Ap), *Aphis glycines* (Ay), *Aphis gossypii* (Ag), *Apis mellifera* (Am), *Bombyx mori* (Bm), *Caenorhabiditis elegans* (Ce), *Drosophila melanogaster* (Dm), *Nilaparvata lugens* (Nl), *Pediculus humanus* (Ph), and *Tribolium castaneum* (Tc).

The phylogenetic analysis of Dcr2 proteins was conducted on the basis of RNAse IIIa domain sequences. Apart from Dcr2 of *D. melanogaster*, insect Dcr2 proteins were placed into 2 clusters (Figure 5A). Interestingly, both aphid Dcr2 proteins (Dcr2 in *A. glycines* and in *A. pisum*) were grouped together with Dcr2 of *T. castaneum*, with the other group including a hymemopteran and a lepidopteran. Using the entire coding region, no evidence of purifying selection was found among insect sequences used (d*N*/d*S* = 0.55, *p* > 0.05).

#### 2.2.2. R2d2

The phylogenetic analysis of various insect R2d2s was largely consistent with the overall phylogeny of different insect orders (Figure 5B). R2d2s belonging to various hemimetabolous and holometabolous insects were clustered together in separate lineages. The R2d2s from *A. glycines*, *A. pisum*, and *P. humanus* clustered together as they represented the paraneoptera. Purifying selection was detected among all species, with the d*N/*d*S* = 0.68 (*p* < 0.001), indicating strong conservation.

#### 2.2.3. Ago2

The phylogenetic analysis conducted on the basis of PIWI domain sequences placed Ago2 proteins of various insects into two clusters (Figure 5C). Ago2 from aphid species (*A. glycines* and *A. pisum*) were grouped along with those of *T. castaneum* and *A. mellifera*. Similar to R2d2, purifying selection was detected among all species, with d*N/*d*S* = 0.51 (*p* < 0.01).

#### 2.2.4. Sid1

Phylogenetic analysis of Sid1 was largely consistent with hemimetabola and holometabola phylogeny of insects (Figure 5D). As a hemimetabolous group, amino acid sequences of Sid1 proteins from 4 hemipteran (*A. glycines*, *A. gossypii*, *A. pisum* and *N. lugens*) and 1 phthirapteran (*P. humanus*) species clustered together. Two Sid1 like proteins in *T. casateneum* (TcSid1a and TcSid1b) and 3 Sid1 like proteins in *B. mori* (BmSid1a, BmSid1b and BmSid1c) formed separate sub-clusters. The sub-clustering of TcSid1c, another Sid1 like protein from *T. casteneum* with that of AmSid1 (from *A. mellifera*) indicates a common lineage for these two. *Sid1* exhibited strong levels of conservation among species with significant evidence of purifying selection (d*N/*d*S* = 0.52; *p* < 0.001).

### 2.3. Expression Analysis of AyDcr2, AyR2d2, AyAgo2, and AySid1

**Figure 6:**
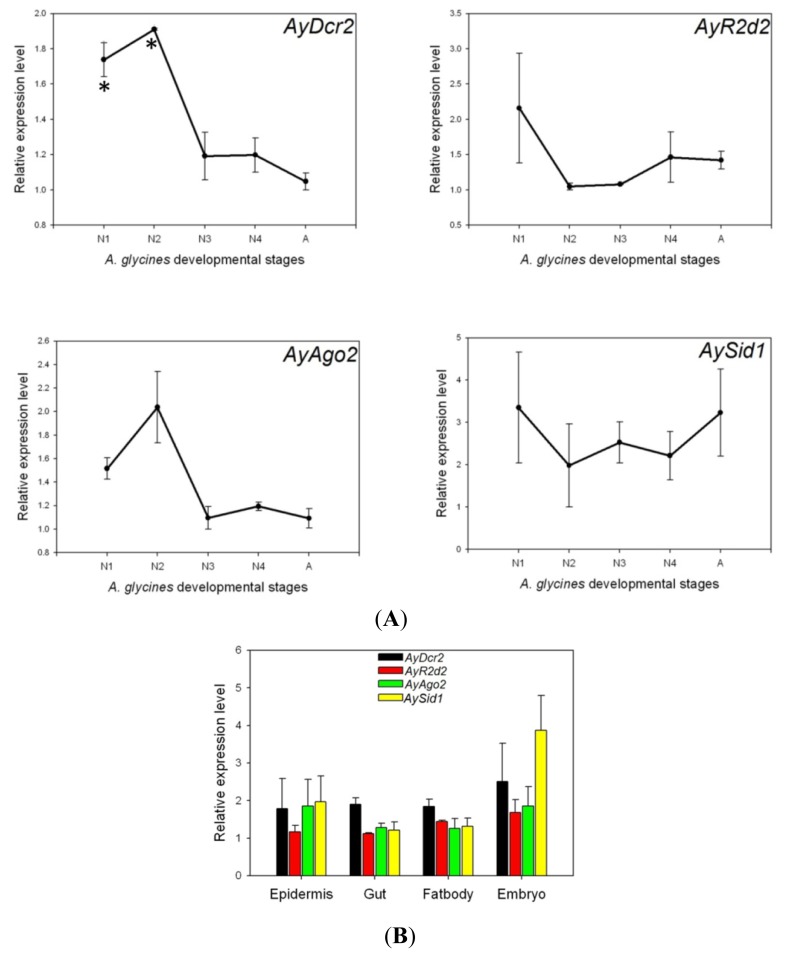
Relative expression levels of *AyDcr2*, *AyR2d2*, *AyAgo2*, and *AySid1* in different developmental stages (**A**) and tissues (**B**) of *A. glycines* as determined by qPCR. The relative expression was calculated based on the value of the lowest expression which was ascribed an arbitrary value of 1. The asterisk (^*^) represents a significant difference (*p* < 0.05) compared to treatment with lowest expression. Different developmental stages are N1-1^st^ instar nymph, N2-2nd instar nymph, N3-3rd instar nymph, N4-4th instar nymph, A-adult. Different tissues dissected from *A. glycines* adults are epidermis, gut, fat body and embryo.

The qPCR analysis indicated that all major genes of RNAi machinery are expressed in every developmental stage of *A. glycines* (Figure 6A). The expression of *AyDcr2* was significantly higher in first and second instar compared to other developmental stages (*p* < 0.05). Though statistically indistinguishable, *AyR2d2* and *AyAgo2* had the highest expression in first and second instar stages of *A. glycines*, respectively. In general, *AyDcr2*, *AyR2d2*, and *AyAgo2* had relatively higher expression in earlier developmental stages (first and second nymphal instar) as compared to later stages (third and fourth instar; and adult). The expression of *AySid1* was nearly identical in all stages. In tissue specific expression analysis, RNAi genes were found to be expressed in all major tissues (epidermis, gut and fatbody) of *A. glycines*, including developing embryos (Figure 6B). Further, qPCR analysis revealed no significant differences in the expression level in these tissues.

## 3. Discussion

In aphids, there is an evidence of extensive gene duplications and positive selection in the miRNA pathway genes, *Dcr-1* and *Ago-1* [20]. In the current study, we did not find any evidence of duplication for RNAi pathway genes (as also reported by [20]), but did observe strong evidence for conservation and purifying selection. The strict selection and conservation seen with RNAi pathway genes may be related to their function in immune response, specifically virus defense. Aphids lack genes involved in the IMD pathway (a signaling pathway in immune response) that functions against viruses [39,40], and bacteria [41] in other insects. In fact, the analysis of *A. pisum* genome has revealed a highly reduced immune system in this insect [41,42], an observation which might extend to other aphids. The role of conservation of RNAi pathway genes is further supported by the ubiquitous expression of these genes in several tissues and developmental stages, which indicate the maintenance of extensively active RNAi pathway (Figure 6). Most aphids, including *A. glycines*, are efficient vectors and are constantly exposed to several plant viruses [43]. As a result of this constant exposure, the RNAi machinery may remain active for virus defense. Further research is needed to fully explain the role of RNAi in virus defense.

Although our sequence homology (Figures 1 and 2) and phylogenetic analyses (Figure 5) suggested conservation in major RNAi genes, this may not correlate with a robust RNAi phenotype in aphids. Previous studies reported only a transient reduction of gene expression in *A. pisum* midgut following dsRNA injection and feeding, respectively [44,45]. In another study, the injection of siRNAs for *coo2*, a gene encoding an aphid salivary protein, resulted in robust knockdown of expression and a lethal phenotype in *A. pisum* [46]. However, a similar response was not observed when *coo2* dsRNA was fed to the green peach aphid, *M. persicae*, through plants [47]. This variation could either be due to a difference in delivery methods or different functions of *coo2* among aphid species. Future studies to knockdown multiple genes in multiple species will better provide the definitive evidence on the RNAi response in aphids. Preliminary attempts in our laboratory using injection have led to significant mortality in control individuals, probably due to the small size of *A. glycines* (about 10 times smaller than the pea aphid). Feeding dsRNA through artificial diet or plants offers the best option for RNAi in this insect. Moreover, RNAi studies through feeding would translate more efficiently in the field for *A. glycines* control using transgenic plants expressing RNAi [48,49]. Although no such feeding assay yet exists, our characterization and gene expression analysis shows that RNAi machinery would unlikely be a limitation.

RNAi experiments in aphid species have been successful [44–47] but it is unknown if these studies led to a systemic response. Among insects that have been investigated so far, *Sid1* has been found in highly diverse insect lineages such as Orthoptera, Phthiraptera, Hemiptera, Coleoptera, Lepidoptera, and Hymenoptera but is absent only in Diptera. In most of these lineages, RNAi has been demonstrated [50]. The presence of *Sid1* in various aphid species [51] tends to support the occurrence of systemic RNAi. The absence of *Sid1* in Diptera is surprising, given our results showing strong conservation and evidence for purifying selection with this gene. Further research into the molecular evolution of *Sid1* should focus on a wide sampling of insect orders, as well as within Diptera, to determine when and how the loss of this gene occurred.

Development and success of RNAi-based transgenic crops for pest control is absolutely dependent upon a robust and systemic RNAi response in target insects. Characterization and comparison of insects’ RNAi machinery coupled with knowledge on their RNAi response may explain the molecular basis of a robust and systemic RNAi. This, in turn, will help to develop methods to derive a robust and systemic RNAi-mediated knockdown in insects. Our study will provide the foundation of future work for controlling one of the most important insect pests of soybean in North America.

## 4. Methods

### 4.1. Identification and Analyses of Dcr2, R2d2, Ago2, and Sid1 cDNAs in A. glycines

To retrieve cDNAs for *Dcr2*, *R2d2*, *Ago2*, and *Sid1* in *A. glycines*, protein sequences of homologs in *T. casteneum* were used as the query in a tblastn search of an *A. glycines* transcriptomic database (Short Read Archive accession: SRX016521, R. Bansal, unpublished data, [33]). In the database, we identified one contig each displaying significant similarity to *Dcr2*, *R2d2*, *Ago2* and *Sid1* homologs in *T. casteneum*. The identity of putative cDNA of *A. glycines* was further confirmed by blastx search at NCBI-GenBank. Based on known insect homologs, cDNA and deduced protein sequences of *AyDcr2*, *AyR2d2*, *AyAgo2*, and *AySid1* appeared to be complete (Note: we have chosen the abbreviation *Ay* to avoid confusion with the *Ag* abbreviation used for genes from *Anopheles gambiae*).The ORF finder tool at National Center for Biotechnology Information (NCBI) internet server was used to identify the open reading frame of putative genes.

Domain architecture of insect Dcr2, R2d2, Ago2, and Sid1 proteins was analyzed by Scan-Prosite [52]. The Scan-Prosite database contains profiles for each available protein. These profiles are in the form of weight-matrix having position-specific amino acid weights and gap costs. To calculate the similarity scores for a protein, amino-acid sequences are compared to profiles available in the database. For Ago2, signature residues in PAZ domain were identified on basis from outlined in [37] whereas the catalytic DDH motif in PIWI domain was identified on the basis outlined in [53].

The transmembrane helices in the protein sequence of *AySid1* were predicted at TMHMM Server (version 2.0; Center for Biological Sequence Analysis: Lyngby, Denmark, 2007) Multiple alignments of various protein sequences were performed by using ClustalW [54,55]. The accession numbers for various protein sequences used in the alignment are provided in the Supplementary Table 1. The *AyDcr2*, *AyR2d2*, *AyAgo2*, and *AySid1* cDNA sequences were deposited in the NCBI GenBank (see Table 1 for accession numbers).

### 4.2. Phylogenetic Analysis of insect Dcr2, R2d2, Ago2, and Sid1

The phylogenetic analysis was conducted in MEGA5.05 software [56]. For phylogenetic analysis, multiple alignments were made from RNAse IIIa domain of Dcr2, both DS_RBDs of R2d2, PIWI domain of Ago2, and full length protein of Sid1 protein. To infer the evolutionary history, the Neighbor-Joining method (with pairwise deletion) was used. A bootstrap test was conducted (100,00 replicates) to calculate the percentages of replicate trees in which sequences clustered together. GenBank accession numbers for various protein sequences used in the phylogenetic analysis are provided in Table S2. Neutrality for each entire gene was determined by testing the null hypothesis that the number of nonsynonymous (d*N*) and synonymous (d*S*) substitutions were equal using the ratio d*N/*d*S*; a value < 1 indicates purifying selection. DNA alignments were performed using MUSCLE [57] in MEGA [56], respective of codons. Overall averages were calculated using the Nei-Gojobori/Jukes-Cantor method [58], using 1000 bootstraps to estimate variance, with complete deletion of gaps and missing data. Rejection of neutrality (d*N/*d*S* = 1) in favor of purifying selection was determined through the test statistic d*S*–d*N*; rejection of the null hypothesis was determined at the 5% level in MEGA.

### 4.3. Insect Culture

*A. glycines* insects were obtained from a laboratory colony, referred to as biotype 1 (B1) that originated from insects collected from Urbana (IL, USA; 40°06′N, 88°12′W) in 2000 [59]. At Ohio Agricultural Research and Development Center (OARDC, Wooster, OH, USA), a laboratory population of these insects is maintained on susceptible soybean seedlings (SD) in a rearing room at 23–25 °C and 15:9 (L:D) photoperiod.

### 4.4. Tissue and Developmental Expression of Dcr2, R2d2, Ago2, and Sid1 in *A. glycines*

To obtain tissue samples (gut, fat body, integument and embryo developing inside adults), *A. glycines* adults (5 days old) were dissected in phosphate buffer saline (pH 8) under a dissection microscope. To determine the expression in different developmental stages, all four nymphal and adult (whole body) samples were collected from insects feeding on susceptible soybean plants (variety SD76R). Total RNA extraction, DNase treatment, cDNA synthesis, and qPCR reactions were performed as described previously [60–62]. Gene-specific qPCR primers were designed using *Beacon Designer* version 7.0 (Palo Alto, CA, USA), and are listed in Table S3. There was no amplification when DNase-treated RNA was used as a template, thus confirming the absence of genomic DNA contamination in RNA samples. To ensure the specificity of qPCR primers, the resultant PCR products were sequenced and their identities were confirmed. The relative expression level for all genes in different tissues and developmental stages was determined by comparative Ct method (2^−ΔCt^) [63]. The significance of differences in the gene expression was determined by *t*-test.

## 5. Conclusions

We have characterized the core components of the RNAi pathway in *A. glycines*, an economically important aphid species. Through comparative genetic analyses among several insect taxa, we show that these components exhibit much conservation across functionally important regions. Additionally, we have identified a putative *Sid1* like cDNA in *A. glycines*, suggesting the presence of a functioning systemic RNAi pathway in this insect, which is a critical first step towards the development of RNAi-based functional genetic assay or novel insect control strategies.

## Figures and Tables

**Table 1:** Description of *Dcr2*, *R2d2*, *Ago2*, and *Sid1* cDNAs in *A. glycines*.

Name	cDNA (bp)	Acc# a	ORF b	EPL (aa) c	MM (kDa) d	p*I*^e^	Match f	%ID g
*AyDcr2*	5877	JX870425	487–5373	1628	187.96	6.23	*Ap*(LOC100166428)	85%
*AyR2d2*	1742	JX870426	139–1155	338	37.88	5.84	*Ap*(LOC100164758)	73%
*AyAgo2*	3372	JX870427	326–3274	982	111.46	9.63	*Ap*(XP_003244047)	78%
*AySid1*	2795	JX870428	138–2438	766	88.39	7.02	*Ag*(EF533711)	99%

a Acc#, Accession number;

b ORF, Open reading frame;

c EPL, Encoded protein length;

d MM, Molecular mass

e p*I*, Isoelectric Point;

f Match, closest homolog: *Ap*-*A. pisum; Ag*-*A. gossypii*;

g %ID, percent identity (at amino acid level) to closest match.

## Supplementary Files

Supplementary File 1 Supplementary Information (PDF, 1296 KB)
