# Supplementary material for: Core RNAi Machinery and Sid1, a Component for Systemic RNAi, in the Hemipteran Insect, Aphis glycines
Source: Int J Mol Sci. 2013 Feb 8;14(2):3786–801. doi: 10.3390/ijms14023786 (PMC3588070; doi:10.3390/ijms14023786)
Supplement: Supplementary File 1 — Supplementary Information (PDF, 1296 KB) [file ijms-14-03786-s001.pdf]

Supplementary Information

Figure S1. Alignment of amino acids sequences from RNAse IIIa (top) RNAse IIIb (bottom) domains of insect Dcr2 proteins. The conserved and similar amino acid residues are labeled in black and grey backgrounds respectively. Red and blue arrowheads indicate catalytic residues of Dcr2 proteins. The figures in the parentheses indicate the percent identity of that protein to the respective domain of AyDcr2. The accession numbers for various protein sequences used in the alignment are provided in Table S2.

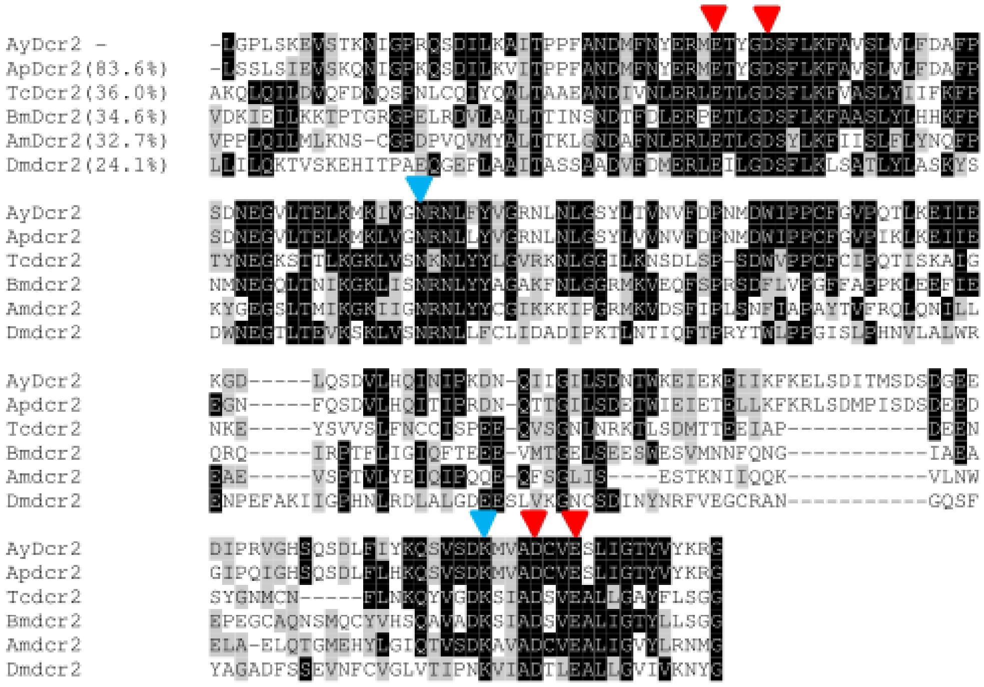

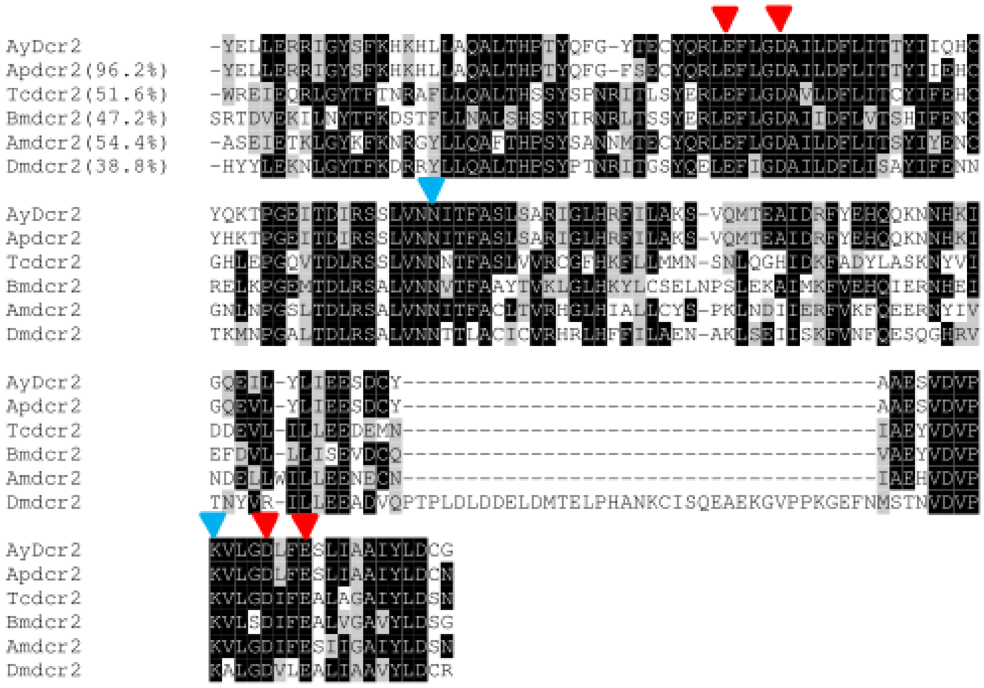

Figure S2. Alignment of amino acids sequences from insect R2d2 proteins. The conserved and similar amino acid residues are labeled in black and grey backgrounds respectively. The accession numbers for various protein sequences used in the alignment are provided in Table S2.

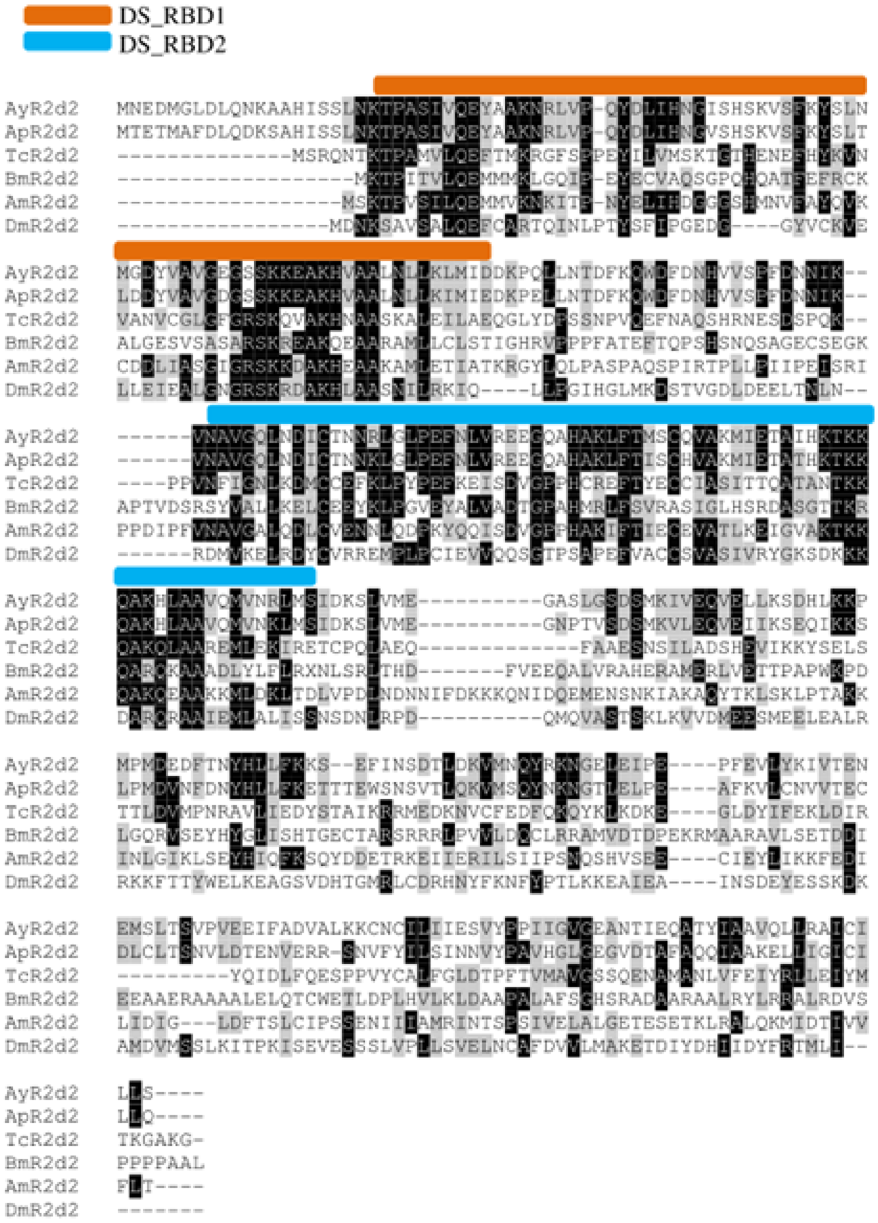

Figure S3. Alignment of amino acids sequences from insect Ago2 proteins. The conserved and similar amino acid residues are labeled in black and grey backgrounds respectively. The accession numbers for various protein sequences used in the alignment are provided in Table S2.

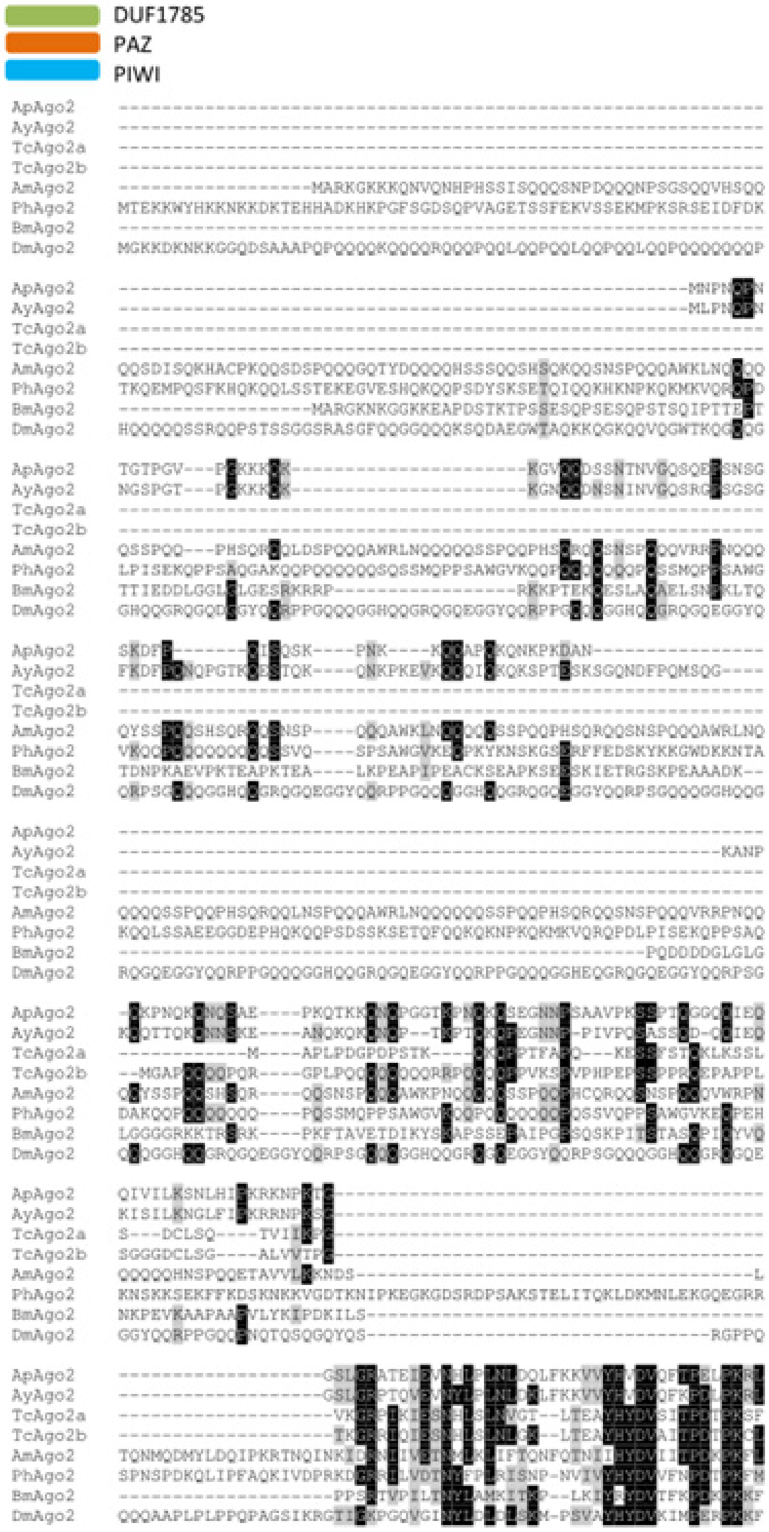

Figure S3. *Cont.*

```
ApAgo2    LRNALEEFNNRHYPKVNFAFDGRRNMYTIKEI----KGKSDTVSVVNDENNRTIDFGIST
AyAgo2    LRNALEEFINKHYPKLLFAFDGRRNMYTTKEI----KGKTDLINVLNDENNRTIDFTITT
TcAgo2a   LRDVMNLFARKHYPKNHPAFDGRKNLYSPKKLPLPNDTMSDTIELEGENKKRG--FKVVV
TcAgo2b   LRDVMNLFGRKHYPQNHPAFDGRKNLYSPKKLPFPNDTKSDTIEVEGENRKKE--FKVEV
AmAgo2    LRTVFEEFRKKQCPKRYPAFDGRKNAYSAKLLPFGDKSKEEEINVFDVNTRKERNFKIYL
PhAgo2    LRLAWNKYASDNFKNRITVFDGKKNAYSKGPLISNGNCHSAGVSIVNPNSGKIREFSVSI
BmAgo2    IAQVFKLVKSKEFPKEILAFDQTKNCYSLTPLPKITTERYGVKVVIKDMNGKDMPFEVSF
DmAgo2    YRQAFEQFRVDQLGGAVLAYDGKASCYSVDKLPLNSQNPE---VTVTDRNGRTLRYTIEI

ApAgo2    SIVNTIHMNKIED-YLKSGSSNTPPGEAFQALDIVLKNRPFALRFTNVGRSFFPVPR--I
AyAgo2    SVVNVIQMNKIED-YLKSGSSNATPGEAFQALDIILKNRPFSLRFTNVGRSFFPLPR--I
TcAgo2a   KLARTVDLSPLR----DILQTRQSPQDALQCLDIVLRNAP-SNSCISSGRCFFTPPRE-G
TcAgo2b   KLARTVDLTPLH----DIMRTTQSPQDALQCLDIVLRNAP-SNACIIAGRCFFTPPRD-G
AmAgo2    NKVACLDLSWLTNLKCDMMDSERN-QKCIQALDIILRHGP-AYQYTVVGRSLFQPPEP-G
PhAgo2    QEVRQDYKQSLEH-------NVKLPEAEISVLEIILKNGP-SNTLVPCGRSFFPKSQMGI
BmAgo2    KASGIVDYNNVLKHMATGGSSLNAPTDTIQCIDIVLKQGT-LESYVKAGRQYFMRPAS--
DmAgo2    KETGDSTIDLKSLTTYMNDRIFDKPMRAMQCVEVVLASPC-HNKAIRVGRSFFKMSDP-N

ApAgo2    TPVDLGEGMELWKGFFQSPVMGWKP-YLNIDVAHKGFPKYQPLINFITNEMN--------
AyAgo2    TPVDLGEGMELWKGFFQSPVMGWKP-FLNIDVAHKGFPKHQPLINYISNELG--------
TcAgo2a   QILRLGDGMEMYYGFYQSAIRGWKQPLLNVDVVHKAFPEALNVLDLVCELGSDYRNTMTR
TcAgo2b   QIIPLGDGMELYYGFYQSAIRGWKA-LLNVDVAHKAFPKASNVLDIVCEIGSDFRTTMTR
AmAgo2    RIVSLSNGLDLWVGVFQSVVIGSKP-YLNIDVAHKGFPKSQSVIELMKELCN-------V
PhAgo2    RARSISSCLDYRAGHYQAATLGDRI-YLNFDLAQKAFYKSLPVIDVAYSFTS--------
BmAgo2    -PIDLGDGLEMWTGLFQSAIFTSKA-FINVDVAHKGFPKNQPMIDAFTRDFR-----LDP
DmAgo2    NRHELDDGYEALVGLYQAFMLGDRP-FLNVDISHKSFPISMPMIEYLERFSLK------A

ApAgo2    CDLNSEMDQRSYNTLASYVKGLKIDFTVPNQPNTK---RSYKVVGLLDTASRFRFEMEDP
AyAgo2    CDLNSPMDNWSFNALSNYVKGLKIDFMVPNQPNTK---RSYKVVGLLDTAAKFRFDMEDP
TcAgo2a   QDLNQPLTDFVQKALEKFLKQLKVTYEIPGQSGSR---RIFRVNGLRAPPSQARFTLGD-
TcAgo2b   ANLSQPLREFVQRDFEKFIKQLKVKYEIPNQSSSK---RIHRVNGLGEPPSQAKFKLDD-
AmAgo2    QDLTPKNIEYNLVNINKFLKGLKIQYELPGQPTSK---RTYRVNKLVDSARENKFRLED-
PhAgo2    ----SYNNRINLDSLNYFLKGAKVEYKLPNSSDPG---KIYKVNGLVGNAEEETFEKDT-
BmAgo2    NRPVDRQPGRAAEAFNEFIRGLKVVSKILGTGPSSGQLREHICNGVVDPPSRQTFTLEND
DmAgo2    KINNTTNLDYSRRFLEPFLRGINVVYTPPQSFQSAP--RVYRVNGLSRAPASSETFEHD-

ApAgo2    VRGKQTLNVVQYFRITRNYVIKHPNLPCLHVGNVNKK--TAIPIELCHVQKGQLRLKKLS
AyAgo2    VRGKQSLNVVQYFKITRNYSIKYQNLPCLHVGNVNKK--TAIPIELCIVQRGQLRLKKLS
TcAgo2a   ---GKVTTVEKYYQEVKRCRLQYPHLPTLWVGSRQRE--VLIPLEFCTVVSGQVVNRKMN
TcAgo2b   ---GRMTTVERYYQEVKRCKLQYPHLPTLWVGSRERESKILLPLEFCTVVGGQAINRKMN
AmAgo2    ---QTLCSVEKYFLQIKKYTIKYPNLPCLWVGSQKNS--IYLPAELCTVIAGQVINKEMN
PhAgo2    ----ITMTVATYFAKDKKYPLKHSHLPLLWVGSTQRK--IYLPMEFCRIVEDQVFMGEMS
BmAgo2    KGPPVRMTVYEYFMKEKKYRIKYPDLNCLWVGPKDKN--IYLPMELVEVAYGQARNKQLN
DmAgo2    ---GKKVTIASYFHS-RNYPLKFPQLHCLNVGSSIKS--ILLPIELCSIEEGQALNRKDG

ApAgo2    EIQTAAMVKNAARPPGERRQTIENCIRDIAYNKDPVLK-DFGIEVKEHFASIPARVLDQP
AyAgo2    EMQTASMVKNAARPPSERRQTIENCIRDIKYNQDPVLN-EFGVNVTEQFASIPARVLDQP
TcAgo2a   ENQTSVMIKKAATSTDVRKDKIMQVLRKANYNSDPCVR-EFGFSVNNSFEKLDGRVLQPP
TcAgo2b   ENQTSAMIRKAATSTDVRKDKIMQTLRTANYNNDPCIR-EFGFSVSNNFEKLDARVLNPP
AmAgo2    KIQTSKMVRETATNTQKRKEKIMNGFAKMNLNQQPTLMNEFHFSVNTEEFEKVPARVLKAP
PhAgo2    PDETREMVKLATSDAKTRKKDILYVFNKANFSNNIYMK-EFGLSVDKDMERIQARILEPP
BmAgo2    DRQLSTMVREAATPPDVRKRKIEEVIQKMNYSKNQFFK-TYGLEIANEFYQVEAKILEAP
DmAgo2    ATQVANMIKYAATSTNVRKRKIMNLLQYFQHNLDPTIS-RFGIRIANDFIVVSTRVLSPP

ApAgo2    SLAYAQNKETKPRAGVWRPDR--FSKAVHINKWVVLNLDQR-----TNIASIKNFEKSLM
AyAgo2    FLAYAQNRETRPRGGVWKPDK--FSKAVHINKWVVLNLDGR-----TNMGSIKNLERNLI
TcAgo2a   TLLYARKAEVTPSKGVWRADMNRFFVGAIVHKWTIVSCTRH-----PER--GEQLADMIF
TcAgo2b   SLLYADNAQIKPSKGVWRADRNRFLVGATINKWTIASGTRY-----PSRD-ADKLADMIF
AmAgo2    KLQYKEK-EITVSKGTWKADKFFSPCVLPKNLWTILNLDKF-----VNAHDLYNLHNKLL
PhAgo2    SLR--TKGSVFVKDGQWKIRD--FFTPTVLDNWCILYFDNC-----VRKENLNDFQNLIK
BmAgo2    TLEVGPRQFTVPKKGVWQANC--LLKPEALNSWGFIAIELD-----PRGCNYEDIVSKLM
DmAgo2    QVEYHSKRFTMVKNGSWRMDGMKFLEPKPKAHKCAVLYCDPRSGRKMNYTQLNDFGNLII

ApAgo2    MSARDLNVVMSPMDPVINIFLPRSS-LADIKTSIGNVFTKQKACNTELIVVVIPDYPAGI
AyAgo2    MSGKELNVVVNPMDPPINCLLQRNMRVAEIKTLVGNNFRKLKQVNTELIVVIIPDAPAGV
TcAgo2a   RMASSNGMQITSKATGPFQHLGGRQNLRDII----DYFK--RKQDHDLIIVVVPNSGP-Q
TcAgo2b   RMASSNGMQITSKAT-PSTHIGGRQGLRDFI----DYFK--GKQDYDLIIVVVPNSGP-Q
```

Figure S3. *Cont.*

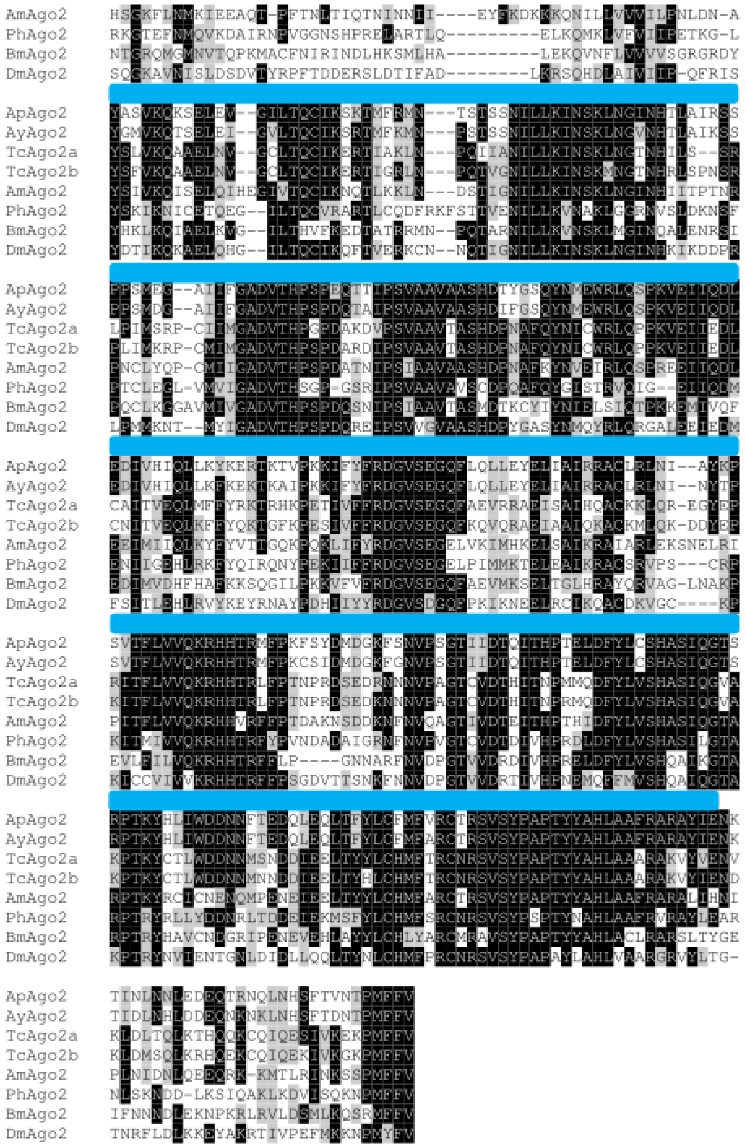

Figure S4. Sid1 protein in *A. glycines*. Italicized and underlined residues at amino-terminal indicate the secretion signal peptide (SignalP). Amino acid residues in black background indicate transmembrane domains (total 11).

<u>*MWKPIFVIIFGLSSVWS*</u>ELFSENVERYGSNDLIPIVLKGNYSQNYPQIINNTMSYLFLYD
YLPNSTFEPPRVKVTLIEPEDNSIVDPLIVVVRHRSGVISWQLPYIEKQQEIKYYKAAHI
LCPLLSASNNESRIVVSVSTNSINNITFILRLDIQKSFNVLLNQEVSFNLSPSEPVYYYY
SFKQNSSMVLLHVKSDDSICMTLSIQNSSCPVFDSLETVQYDGLRQTVSKTGGIIISKDE
YPLGLFIVFVVHSDDSACHQGNYQAINFRTKSINFVVKPTVDFNYQIINCLIVIVIFIFI
LVFTTFFYHTKGDDLKTIDIIQEEPSTSICSTPVNIQPDNVSYDSSLDETDIDILKSPEP
WKDLIRTKACLYVSDLSKKDHRILKAKSRLYVWNLITVAVFYSLPVTQLVFTSQKMLIET
GNQDLCYYNFLCSHSFILGPWKFSDFNHIFSNIGYIFFGLLFILITYKRECVNIPNKKFG
IPNHYGLYYAMGSALAMEGLMSACYHVCPNHSNFQFDTSFMYVICMLSMIKIYQTRHPDI
NANAYLVFGVLALVIILGLTGIMYEGPILFVLFTCLHLIMIFWLSAQIYYMGRWKLDKKT
PKRFLNHIMTAPNPCRPKYPNRMVLLSFGILINLGLAVSHWIIKFGNFGNYLLILFMVNL
ILYLSFYIVMKLISKEKLHFWPLLYILLAVIFWSASLYFYVHKSSSWTLSAAESRTYNTP
CTFMDFYDNHDIWHFLSAISLFLSFMVLFTLDDDVNSKPTATIPVF

Figure S5. Alignment of amino acids sequences from insect Sid1 proteins. The conserved and similar amino acid residues are labeled in black and grey backgrounds respectively. The accession numbers for various protein sequences used in the alignment are provided in Table S2.

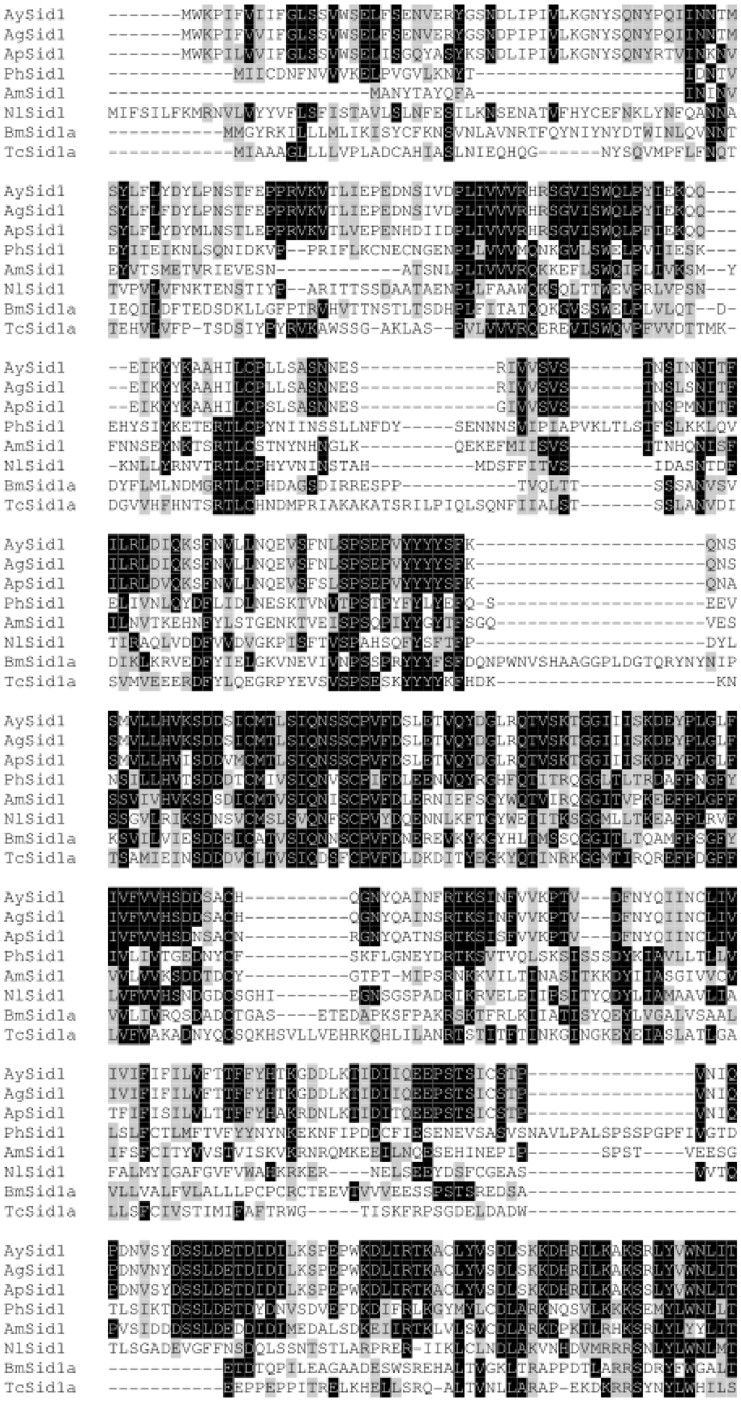

Figure S5. *Cont.*

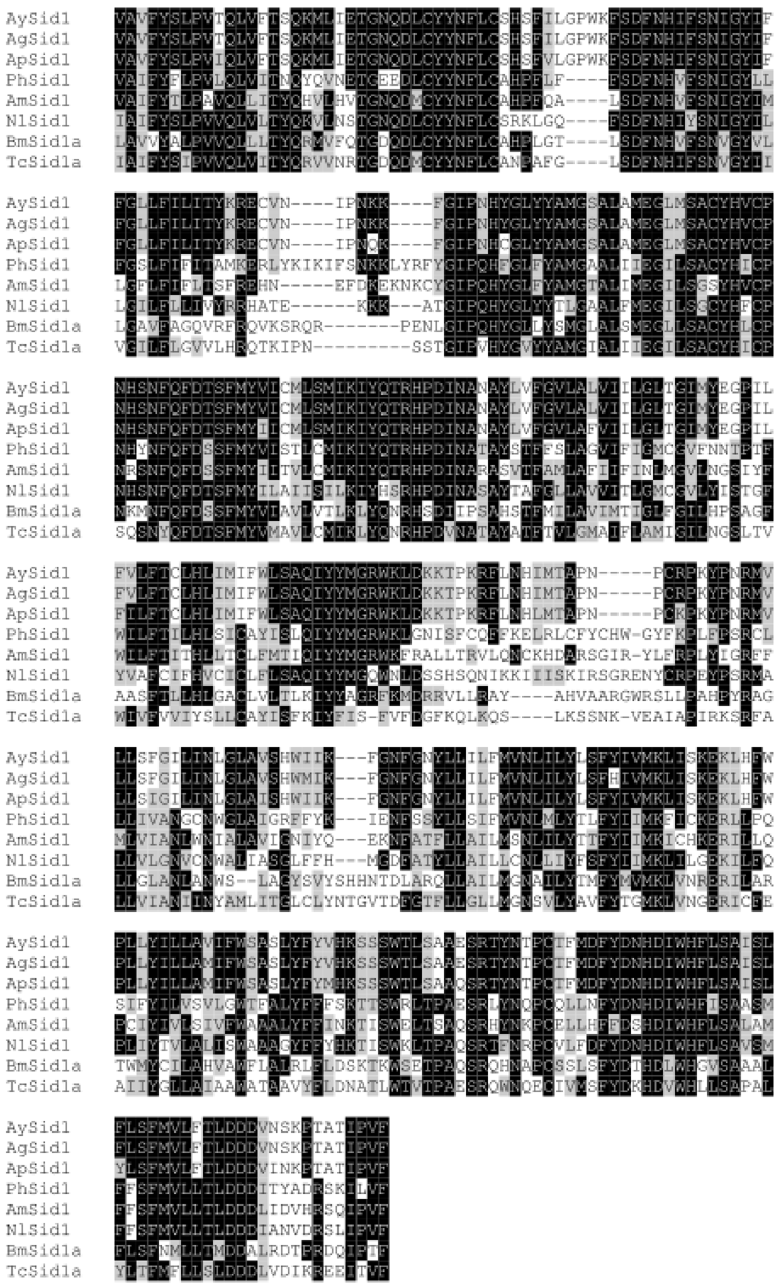

Table S1. Scanprosite hit scores for various domains in insect Dcr2 proteins.

Domains	Dcr2 proteins				
	**AyDcr2**	**ApDcr2**	**TcDcr2**	**BmDcr2**	**DmDcr2**
Helicase (a)	19.5	19.7	22.2	21.4	18.7
Helicase (b)	14.0	13.6	12.1	12.6	12.1
Dicer_DSRBF	27.0	26.1	24.8	23.5	24.9
PAZ	17.3	18.4	17.2	11.9	8.7
RNase III (a)	20.1	19.6	23.9	21.3	18.4
RNase III (b)	35.5	34.0	36.9	34.7	31.1
DSRBD	10.0	9.6	-	9.1	9.7

Table S2. GenBank accession numbers for various protein sequences used in various analyses.

Name	Accession #
ApDcr2	XP_003240110.1
AmDcr2	XR_120636.1
BmDcr2	NP_001180543.1
CeDcr2	NP_498761.1
DmDcr2	NP_523778.2
TcDcr2	NP_001107840.1
ApR2d2	NP_001155644.1
AmR2d2	XP_001121349.2
BmR2d2	NP_001182007.1
CrRde4	NP_499265.1
TcR2d2	NP_001128425.1
DmR2d2	NP_609152.1
PhR2d2	XP_002430935.1
ApAgo2	XP_001944852.2
AmAgo2	XP_395048.4
BmAgo2	NP_001036995.2
CeAgo2	NP_871992.1
DmAgo2	NP_648775.1
PhAgo2	XP_002422648.1
TcAgo2a	NP_001107842.1
TcAgo2b	NP_00107828.1
AgSid1	ABP98803.1
ApSid1	XP_001951907.1
AmSid1	XP_395167.4
BmSid1a	NP_001106735.1
BmSid1b	BAF95807.1
BmSid1c	NP_001106736.1
CeSid1	NP_504382.2
NlSid1	ADI88514.1
PhSid1	XP_002430777.1
TcSid1a	NP_001099012.1
TcSid1b	NP_001103253.1
TcSid1c	NP_001099128.1

Table S3. qPCR primer sequences and product length.

Name	Primer sequences	Product length (bp)
AyDcr2	GAGATTTCCTGACTCTTTAGC<br>CCGATTGCCGAATAACAA	100
AyR2d2	AATCTGAAGACGCACATC<br>TAGCCGCATACTCTTGTA	135
AyAgo2	GAATCGTCAATCGTCATT<br>AACAGAATACTCAGAATATCG	93
AySid1	ACCATCAGAACCAGTGTATT<br>TGTCGTAAGCCATCATATTGTA	165

© 2013 by the authors; licensee MDPI, Basel, Switzerland. This article is an open access article distributed under the terms and conditions of the Creative Commons Attribution license (http://creativecommons.org/licenses/by/3.0/).
